# A proteomic network approach resolves stage-specific molecular phenotypes in chronic traumatic encephalopathy

**DOI:** 10.1186/s13024-021-00462-3

**Published:** 2021-06-25

**Authors:** Laura Gutierrez-Quiceno, Eric B. Dammer, Ashlyn Grace Johnson, James A. Webster, Rhythm Shah, Duc Duong, Luming Yin, Nicholas T. Seyfried, Victor E. Alvarez, Thor D. Stein, Ann C. McKee, Chadwick M. Hales

**Affiliations:** 1 Center for Neurodegenerative Disease, Emory University School of Medicine, 615 Michael Street, Office 505H, Atlanta, GA 30322 USA; 2 Department of Neurology, Emory University School of Medicine, Atlanta, GA 30329 USA; 3 Department of Biochemistry, Emory University School of Medicine, Atlanta, GA 30322 USA; 4 Boston University Alzheimer’s Disease and CTE Center, Boston University School of Medicine, 72 East Concord St, Boston, MA 02118 USA; 5 Department of Neurology, Boston University School of Medicine, 72 East Concord St, Boston, MA 02118 USA; 6 Department of Pathology and Laboratory Medicine, Boston University School of Medicine, 72 East Concord St, Boston, MA 02118 USA; 7 VA Boston Healthcare System, 150 S Huntington Ave, Boston, MA 02130 USA; 8 Department of Veterans Affairs Medical Center, 200 Springs Rd., Bedford, MA 01730 USA; 9 Department of Anatomy and Neurobiology, Boston University School of Medicine, 72 East Concord St, Boston, MA 02118 USA

**Keywords:** Chronic traumatic encephalopathy (CTE), Tandem mass tagged (TMT), Proteomics, Frontotemporal dementia (FTD), Immunoglobulin, Weighted gene co-expression network analysis (WGCNA), Astrocyte

## Abstract

**Background:**

There is an association between repetitive head injury (RHI) and a pathologic diagnosis of chronic traumatic encephalopathy (CTE) characterized by the aggregation of proteins including tau. The underlying molecular events that cause these abnormal protein accumulations remain unclear. Here, we hypothesized that identifying the human brain proteome from serial CTE stages (CTE I-IV) would provide critical new insights into CTE pathogenesis. Brain samples from frontotemporal lobar degeneration due to microtubule associated protein tau (FTLD-MAPT) mutations were also included as a distinct tauopathy phenotype for comparison.

**Methods:**

Isobaric tandem mass tagged labeling and mass spectrometry (TMT-MS) followed by integrated differential and co-expression analysis (i.e., weighted gene co-expression network analysis (WGCNA)) was used to define modules of highly correlated proteins associated with clinical and pathological phenotypes in control (*n* = 23), CTE (*n* = 43), and FTLD-MAPT (*n* = 12) post-mortem cortical tissues. We also compared these findings to network analysis of AD brain.

**Results:**

We identified over 6000 unique proteins across all four CTE stages which sorted into 28 WGCNA modules. Consistent with Alzheimer’s disease, specific modules demonstrated reduced neuronal protein levels, suggesting a neurodegeneration phenotype, while other modules were increased, including proteins associated with inflammation and glial cell proliferation. Notably, unique CTE-specific modules demonstrated prominent enrichment of immunoglobulins, including IGHM and IGLL5, and extracellular matrix (ECM) proteins as well as progressive protein changes with increasing CTE pathologic stage. Finally, aggregate cell subtype (i.e., neurons, microglia, astrocytes) protein abundance levels in CTE cases were similar in expression to AD, but at intermediate levels between controls and the more exaggerated phenotype of FTLD-MAPT, especially in astrocytes.

**Conclusions:**

Overall, we identified thousands of protein changes in CTE postmortem brain and demonstrated that CTE has a pattern of neurodegeneration in neuronal-synaptic and inflammation modules similar to AD. We also identified unique CTE progressive changes, including the enrichment of immunoglobulins and ECM proteins even in early CTE stages. Early and sustained changes in astrocyte modules were also observed. Overall, the prominent overlap with FTLD-MAPT cases confirmed that CTE is on the tauopathy continuum and identified CTE stage specific molecular phenotypes that provide novel insights into disease pathogenesis.

**Supplementary Information:**

The online version contains supplementary material available at 10.1186/s13024-021-00462-3.

## Background

Although age is the greatest risk factor for neurodegenerative disorders like Alzheimer’s disease (AD), there are other possible environmental contributors including prior exposure to traumatic brain injury (TBI) [1–4]. Unfortunately, we do not understand the basic mechanisms that link TBI to neurodegeneration. Studies are often complicated by a heterogenous mix of head injuries as well as a prolonged time period between head injury and the onset of cognitive symptoms, and these challenges have impacted our ability to identify biomarkers and potential therapeutic targets [4, 5]. One form of TBI, repetitive concussive and sub-concussive head impacts (RHI), is associated with postmortem neuropathological changes of chronic traumatic encephalopathy (CTE) [3, 6, 7]. Since CTE involves increasing pathological burden associated with greater RHI and increased age and because we previously identified novel protein changes in neurodegenerative disorders [8–12], including CTE [13], we hypothesized that quantitative proteomics and bioinformatics analysis of molecular changes in CTE brain could provide critical new insights into disease pathogenesis.

Neuropathological studies previously identified important CTE-related changes, including the prominent aggregation of hyperphosphorylated tau around vasculature and deep within the sulci [3, 6, 14]. Tau aggregations are found in both neurons and glial cells, while other pathologic accumulations including TAR DNA binding protein 43 (TDP43), Lewy bodies, and occasionally beta amyloid are also found. Staging criteria for the p-tau pathologic burden in CTE has also been proposed (CTE I-IV), suggesting that there is an orderly progression to the spread of p-tau and potentially other biomarkers of disease [3, 6, 15]. Similarly, there is an association with years of RHI exposure and age with the CTE stages (i.e., CTE I cases are younger and with increasing RHI and age, there is an increase in the CTE stage) [16]. There is also increased risk for CTE with early play and injury, even prior to adulthood [17]. Understanding the molecular changes that occur with increasing CTE stage is feasible since pathological hallmarks, like tau, demonstrate a progressive phenotype [15].

We recently described changes in the CTE insoluble brain proteome in CTE cases with increasing stage [13]. The insoluble proteome contains a smaller subset of proteins prone to aggregate within the brain in neurodegenerative diseases. When compared to controls, we identified differentially expressed proteins in CTE including NADPH quinone oxidoreductase (NQO1), a protein involved in managing reactive oxygen species in the brain and localized to glial cells, including astrocytes with hyperphosphorylated tau. Higher levels of NQO1 also correlated with higher CTE stage, and concordantly, p-tau pathological burden. Similarly, proteomics on a small cohort of CTE cases recently identified downregulation of axonal proteins in stage IV CTE cases, suggesting that specific effects on axons may contribute to the disease process [18]. In an orthogonal approach, ribonucleic acid (RNA) sequencing of CTE cases also demonstrated disease related changes, including alterations in kinases, phosphatases and calcium signaling pathways that may impact tau hyperphosphorylation and disease pathogenesis [19, 20]. Although the proteomic and RNA-seq studies utilized brain samples from a cross-sectional retrospective cohort, the results suggested that a larger proteome study might afford novel insights into CTE pathogenesis.

To provide a more comprehensive characterization of the CTE brain proteome and to identify additional novel biomarkers of disease, therapeutic targets and potential molecular underpinnings, we performed quantitative mass spectrometry utilizing isobaric tandem mass tags (TMT) and off-line fractionation to identify proteins that associated with increasing CTE stage. Integrated differential expression and weighted co-expression network analysis (WGCNA) was then used to identify CTE-specific modules of co-expressed proteins related to biological pathways and cell-type. We also correlated results with other neurodegenerative diseases, including AD and a related tauopathy, frontotemporal lobar degeneration with microtubule associated protein tau mutations (FTLD-MAPT), to identify neurodegenerative disease-related changes.

## Methods

### Collection of human postmortem brain

Brain tissues were obtained from the Emory Goizueta Alzheimer’s Disease Research Center Neuropathology Brain Bank in Atlanta, Georgia, and from the Veterans Affairs-Boston University Concussion Legacy Foundation (VA-BU-CLF) brain bank in Boston, Massachusetts. Brains were collected from deceased subjects after obtaining consent from family under Institutional Review Board (IRB)-approved protocols. Brains were divided into hemispheres to allow for one hemisphere to be coronally sectioned and stored at -80C while the other hemisphere was fixed for immunohistochemistry. Samples of lateral prefrontal and temporal lobe cortex from fresh frozen brain were collected for the current proteomics study. The research subjects used in this study were selected based on demographics and underlying neuropathological diagnosis, which was previously determined with detailed neuropathological characterization to assess for neurodegenerative disease-associated pathologies (Table S1). This included standard immunohistochemistry methods with hematoxylin and eosin as well as silver stain. More targeted immunohistochemistry included labeling for amyloid beta 42, hyperphosphorylated tau, alpha-synuclein, and phosphorylated TDP43 was also used. Evaluations were performed by board-certified neuropathologists. CTE cases were staged using the McKee staging scheme [3, 15]. FTLD-MAPT cases were identified through genetic sequencing of the MAPT gene or characteristic pathology. A demographic summary is also provided in Table S1. CTE cases were all male based on the samples available at the time the project was initiated. The average age of early stage CTE cases was also younger, as noted previously [21].

#### Preparation of brain homogenates

Brain homogenates were prepared from lateral prefrontal and temporal lobe cortex as previously described [22]. Briefly, ~ 100 mg of frozen brain was homogenized in 500 μl of 8 M urea lysis buffer with protease inhibitors (8 M urea, 100 mM NaHPO_4_, pH 8.5; HALT phosphatase/protease inhibitor (Thermo Fisher, Catalog #78440)). A Bullet Blender (Next Advance) was used to homogenize the tissues in 1.5 ml Rhino Tubes with stainless steel beads (2 × 5 min at 4C). Samples were transferred to clean 1.5 ml Eppendorf tubes, sonicated 3–4 times 15 s with 5 s between, and centrifuged at 1000×g. Protein assay (bicinchonic acid; BCA) was conducted and 25 μg was loaded on a sodium dodecyl sulfate polyacrylamide gel electrophoresis (SDS-PAGE) to check integrity of the samples. One hundred micrograms protein was digested with lysyl-endopeptidase and trypsin followed by TMT labeling as previously described. A global internal standard (GIS) was also generated by mixing a small amount from each sample, and the GIS was used to normalize abundances. Samples were first randomized into groups of 9 for TMT labeling and each group also contained two GIS samples to flank the 9 samples in each batch during the proteomic run. TMT labeling kits were made for 11 samples, hence the 9 samples plus 2 GIS. A total of 10 TMT kits were used to generate 10 batches in preparation for liquid chromatography and mass spectrometry.

#### Liquid chromatography

ERLIC fractionation was performed as previously described [23]. Briefly, TMT-labeled peptides were dissolved in 100 μL of 80% (v/v) loading buffer (10 mM NH_4_Ac, 85% ACN/1% acetic acid), injected completely with an auto-sampler, and fractionated using a PolyWAX LP anion-exchange column (200 × 3.2 mm, 5 μm, 300 Å; PolyLC, Columbia, MD) on an Agilent 1100 HPLC system monitored at 280 nm. Forty fractions were collected with a 66-min gradient of 100% mobile phase A (90% ACN/0.1% acetic acid) for 3 min, 0–20% mobile phase B (30% ACN/0.1% FA) for 50 min, 20–100% B for 5 min, followed by 8 min at 100% B at a flow rate of 0.3 ml/min. The 40 fractions were pooled into 20 fractions.

### Tandem mass spectrometry (LC-MS/MS)

#### Tandem mass spectrometry (LC-MS/MS) Lumos Batches

Peptide fractions were reconstituted in 50ul of loading buffer (0.1% formic acid and 0.03% trifluoroacetic acid in water) and 2ul (~2μg) was loaded onto in-house packed 70 cm long 75 um ID column and eluted using a Water’s NanoAcquity operating at a rate of 200 nl per min over 190 mins (load time included). The gradient started from 1% B (0.1% formic acid in acetonitrile) to 35% B over 165 mins. This was followed by a 10 wash at 99% B and finally an equilibration for 15 mins back at 1% B. The Fusion Lumos mass spectrometer was set to collect at top speed for 3 s cycles. Each cycle consisted of 1 full survey scan (120,000 resolution, scan range 380–1500, automatic gain control (AGC) at 200,000 and 50 ms maximum injection time) followed by ion trap CID tandem scan (0.7 m/z isolation window, 35% collision energy, 10,000 AGC and 50 ms maximum injection time) and paired Orbitrap synchronous precursor selection MS3 scans (50,000 resolution, 65% collision energy, 100,000 AGC and 105 ms maximum injection time).

#### Tandem mass spectrometry (LC-MS/MS) Fusion Batches

Peptide fractions were reconstituted in 50ul of loading buffer (0.1% formic acid and 0.03% trifluoroacetic acid in water) and 2ul (~2μg) was loaded onto in-house packed 70 cm long 75 um ID column and eluted using a Dionex RSLCnano operating at a rate of 250 nl per min over 160 mins (load time not included). The gradient started from 1% B (0.1% formic acid in acetonitrile) to 35% B over 165 mins. This was followed by a 10 wash at 99% B and finally an equilibration for 15 mins back at 1% B. The Fusion mass spectrometer was set to collect at top speed for 5 s cycles. Each cycle consisted of 1 full survey scan (120,000 resolution, scan range 380–1500, automatic gain control (AGC) at 200,000 and 50 ms maximum injection time) followed by ion trap CID tandem scan (0.7 m/z isolation window, 35% collision energy, 10,000 AGC and 50 ms maximum injection time) and paired Orbitrap synchronous precursor selection MS3 scans (60,000 resolution, 65% collision energy, 100,000 AGC and 120 ms maximum injection time).

### Proteomic data analysis

Raw data files from the Orbitrap Fusion and Lumos were processed as previously described using Proteome Discoverer [22]. Briefly, Proteome Discoverer (ThermoFisher Scientific version 2.2.0.388) was used to search all rawfiles (Synapse ID: syn11612204; ProteomeXchange ID: PXD024724). The spectra were searched against a human database (90,304 target sequences) downloaded April 2015. Search parameters included 20 ppm precursor ion tolerance, 0.6 Da fragment ion tolerance, static modification for carbamidomethyl cysteines (+ 57.021 Da) and TMT-labeled peptide n-terminals and lysines (+ 229.163 Da), dynamic modifications for oxidized methionines (+ 15.995 Da) and deamidated asparagine and glutamine (+ 0.984 Da). PSM and Peptide level data were filtered to 0.01% FDR. Strict parsimony was applied for protein grouping. TMT reporter ions were matched with a 20 ppm tolerance window and only razor and unique peptides were considered for quantitation. The UniProtKB Human proteome database was used to identify the MS/MS spectra. Peptides were then assembled into proteins to determine abundances based on extracted ion intensities and gene symbol redundancy was managed as previously described (Table S5) [22]. Data was normalized using the GIS samples and only proteins identified all of the 10 batches were included.

ComBat (R sva package) was utilized to remove variability due to batch, due to the two mass spectrometer platforms utilized, and due to the two different brain regions of case samples and ultimately increased the power of our analysis as described here. First, only 6713 well-quantified proteins with no missingness across all 10 TMT batches were considered. Specifically, this process began with removal of WGCNA network connectivity outliers (|Z.k| > 2 SD from mean) within abundance data collected on each of the two LC-MS/MS platforms separately. Then, the matrix of Proteome Discoverer normalized abundance of TMT reporters was split to arrive at 2 input matrices of data, one per LC-MS/MS platform. ComBat was first run separately on each of these two platform-specific matrices to remove TMT batch effects modeling explicitly diagnosis and region; then, the two platform data were combined and, using the same model, ComBat was used to address platform effects (batch was specified as MS/MS platform 1 or 2). Then GIS samples, which were technical replicates of all representative samples across batches and which were strong anchors for ComBat elimination of cross-batch variance were removed. A fourth and final ComBat pass was run, explicitly modeling only sex, and the two brain regions of samples (temporal or frontal) were considered as the desired batch effect to remove in order to arrive at region-agnostic profiles of protein abundance. The use of ComBat for removal of region specific effects in proteomic data was previously used by our group [11], and doing so increases power to discern differences agnostic to region. We used the variancePartition package in R to determine and visualize the improvement in covariance with unwanted factors (Fig. S1).

Following this, nonparametric bootstrap regression removed age and PMI covariance according to a linear mixed model that also included (protected) diagnosis or CTE stage. Fully covariate and regressed abundances were used for all downstream analyses including differential expression and WGCNA. Differential abundance was calculated by subtracting average control protein abundance from disease protein abundance, with one-way ANOVA plus Tukey’s honestly significant difference (Tukey HSD) to determine statistical significance (Table S2). Gene-ontology pathway analysis was run on module-level collections of gene products (Fig. S6), and as there are far fewer modules than genes, we accepted the terms determined with a Z-score greater than 1.96 (*p* < 0.05). Enrichment was calculated with a hypergeometric test and FDR was calculated with Benjamini-Hochberg procedure for multiple comparisons using GO-Elite and string-db.org. WGCNA was performed as previously described [11] using protein abundances to generate modules and identify kME values for module membership of proteins (Table S4). Briefly, the power at which approximate scale free topology was determined at the elbow of the curve of power vs. R-squared approaching an asymptote, ideally above R^2^ = 0.8 and with overall network connectivity reduced to around 100; this power was 7.0 for the regressed data. Processing all data in a single block, modules were merged conservatively in the blockwiseModules function using a mergeCutHeight of 0.07 and reassignThresh = 0.05 (instead of the default 1e-6); other parameters included PAMstage = TRUE, pamRespectsDendro = TRUE, replaceMissing = TRUE, a minimum module size of 17 (per [11] deepSplit = 2, corType = “bicor”, and networkType = “signed”, in addition to TOMDenom = “mean”.

Cell subtype analysis was performed as previously described [22] to identify specific WGCNA modules enriched with marker proteins of specific cell types in the brain using both a reference cell subtype proteome [24] and transcriptome [25]. The biomaRt package (www.biomart.org) and getLDS function was used to convert gene symbols between human and mouse using a comprehensive ensemble database lookup. The digital sorting algorithm (DSA)::EstimateWeight R package and function, with method parameter set to “LM” (i.e., linear modeling), were used to calculate sample-wise five proteome-derived [24] cell type weights or proportions of each sample [26]. Significance of changes across diagnosis-defined groups for modules and cell subtype analyses, independent of age, sex, and PMI effects, was then calculated as a Kruskal-Wallis *p*-value using the R pf function applied to F-statistics. These were arrived at by summarization of the linear model of weights across samples after explicitly considering diagnosis group, age, sex, and PMI.

We also generated a synthetic eigenprotein for AD proteins within the CTE modules to provide a visual comparison between the two data sets (i.e. CTE and AD [22]). Briefly, protein module members in the AD network with a kME in the top 10th percentile were assembled into a synthetic module within the CTE network. Synthetic modules with at least 4 members were used to calculate synthetic weighted eigengenes representing the variance of all members in the target network across case samples via the WGCNA::moduleEigengenes() function. Boxplots of the AD synthetic eigengenes were generated and plotted adjacent to the CTE eigengenes and Kruskal-Wallis *p*-values were calculated.

### Protein blotting and quantitation

To validate select proteomic targets, we utilized standard protein electrophoresis and blotting techniques as previously described [8]. Briefly, 10-20 μg of brain homogenate was combined with 4x sample buffer (ThermoFisher) and loaded in precast 17-well SDS 4–20% gels. Following electrophoresis, proteins were transferred from the gel to a nitrocellulose membrane using the ThermoFisher iBlot system. Membranes were blocked, incubated with primary antibody (against IGHM, IGLL5, GFAP, HEPACAM, GAPDH), fluorescent secondary antibody and then imaged on a LI-COR Odyssey Scanner. Standard washes (3x) in TBS were utilized between incubation steps. ImageJ, Image Studio Lite and Prism was utilized to quantify densitometry of bands and for statistics. Values were normalized to GAPDH. Student’s T test or one-way ANOVA was utilized.

## Results

### TMT labeling and LC-MS/MS provide deep CTE proteomic quantification

**Fig. 1:**
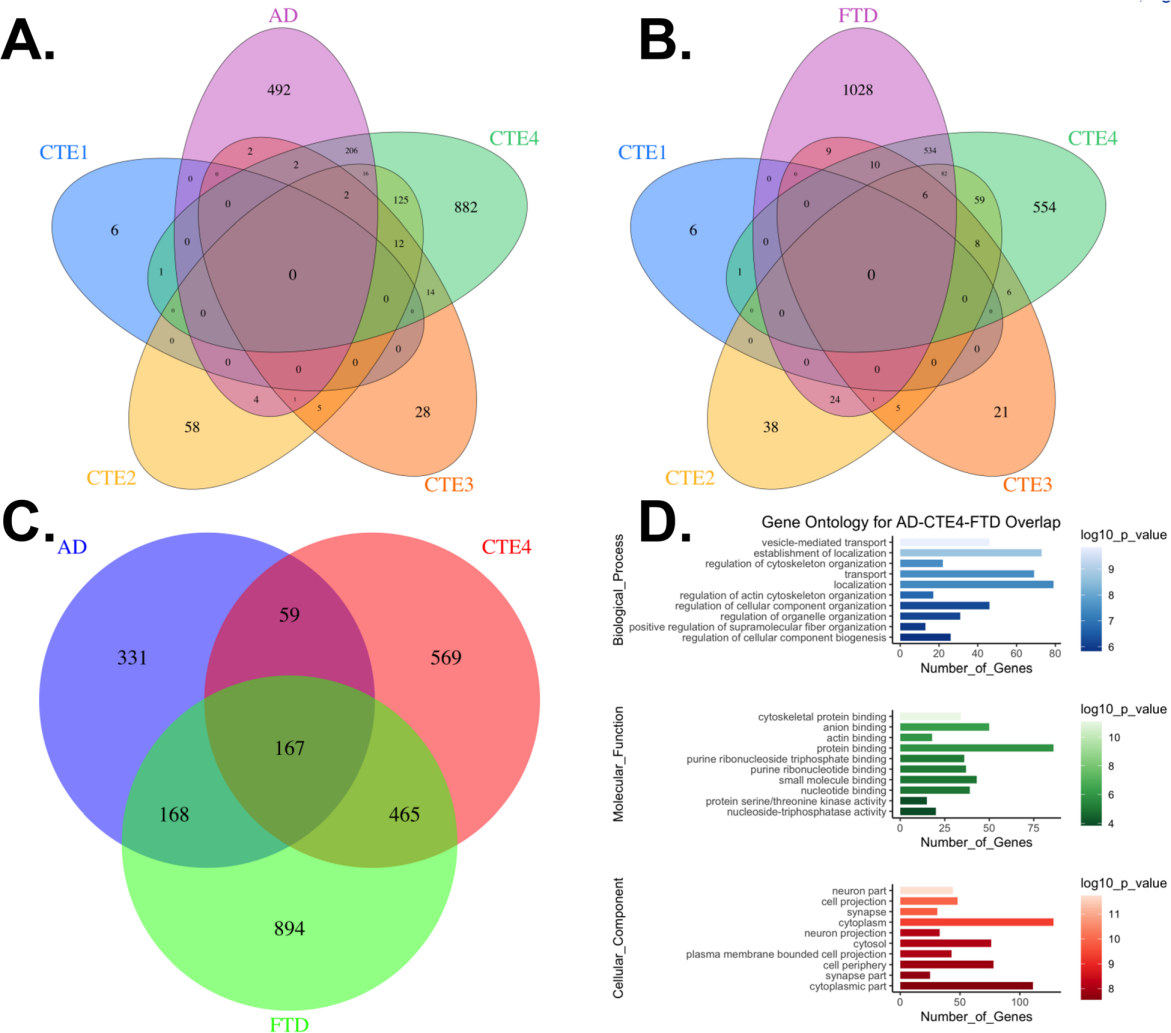
**Overlap of significant differentially expressed proteins in CTE, FTLD-MAPT, and AD as compared to controls**. Proteins were grouped within disease state and sorted based on *p*-value< 0.05 (ANOVA). Venn diagrams showing 5 way overlap between A. AD or B. FTD and CTE I-IV. C. Venn diagram showing 3 way overlap between CTE IV, AD, and FTLD-MAPT. D. Gene ontology showing Biological Process, Molecular Function and Cellular component for 167 proteins significantly enriched in all 3 neurodegenerative disorders as compared to controls

We previously utilized LC-MS/MS to identify significantly enriched proteins in the CTE insoluble proteome [13]. This allowed us to find proteins that were prone to aggregation. Since that study, our group has utilized TMT labeling with off-line fractionation to increase the proteome depth in the human brain [22, 27]. TMT labeling was used to compare controls to symptomatic and asymptomatic AD (AsymAD) in order to identify changes that might be involved in AD progression [22]. Along these same lines, we hypothesized that this technique would generate deep coverage of the CTE total proteome and provide insights into proteins that change with CTE progression compared to controls. Furthermore, we compared CTE to other tauopathies, including frontotemporal lobar degeneration due to MAPT mutations (FTLD-MAPT), progressive supranuclear palsy (PSP), and corticobasal degeneration (CBD). We utilized control (*n* = 22), CTE1 (*n* = 6), CTEII (*n* = 11), CTEIII (n = 11), CTEIV (*n* = 15), FTLD-MAPT (*n* = 12), PSP (*n* = 7) and CBD (n = 2) samples for this proteomic study (Table S1). From a total of 87 samples across 10 TMT batches, LC-MS/MS identified over 10,000 unique proteins (Table S5). When restricted to proteins without any missing values across all 10 batches and after outlier removal, this number dropped to 83 samples and 6713 unique proteins identified across the CTE stages and other tauopathies, nearly double that found in previous studies [22]. Based on differential expression and ANOVA-Tukey HSD significance (*p* < 0.05), we identified 7 (I), 223 (II), 66 (III), and 1260 (IV) differentially expressed proteins across the CTE stages, respectively compared to controls (Table S2, Fig. S2). For comparison, we likewise identified 1694 differentially expressed proteins in FTLD-MAPT, 77 in PSP, 41 in CBD and 725 in AD [22] compared to controls. We also determined the number of significant proteins present in both CTE and AD, given the previously described neuropathological overlap [14], as well as CTE and FTD (Fig. 1) to understand overlap with related tauopathies. Overlap with CTE I-III and AD or FTD was limited, however we identified 226 significant proteins common to CTE IV and AD and interestingly, we identified 632 common to CTE IV and FTD. Gene ontology analysis defined biological processes, molecular function and cell components in the group of 167 proteins common to CTE IV, AD and FTD (Fig. 1), including proteins associated with vesical mediated transport, cytoskeletal organization, synapse and other neuronal components.

### Cortex of CTE neuropathologic stages sits on a continuum between control and FTLD-MAPT as observed with weighted co-expression network analysis

**Fig. 2:**
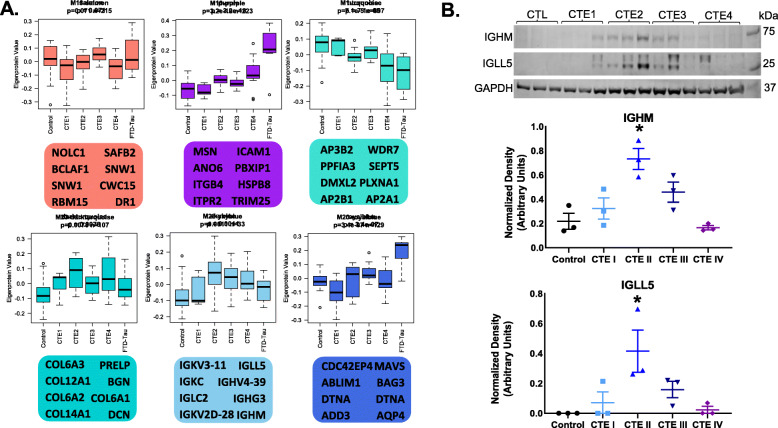
**WGCNA modules demonstrated significant disease-related changes, including enrichment of immunoglobulins in CTE**. We generated 28 WGCNA modules from the control, CTE, and FTLD-MAPT proteomic data. **A**. Representative modules are shown with the top 8 module hub proteins (based on kME value; Table S4) listed below each box plot. Kruskal-Wallis *p*-values for nonparametric ANOVA following linear modeling of covariates and diagnostic groups are shown in above the box plots along with the number (n) of proteins in each module (module sizes also shown in Table S6). **B**. (Top): Protein blot of IGHM and IGLL5, key hub proteins in the M28-skyblue module, in control and CTE I-IV as a validation of the proteomic data. GAPDH shown as a loading control. B. (Bottom): Box plots of protein blot densitometry for IGHM and IGLL5. *designates p-value < 0.05 for CTE II cases using ANOVA (IGHM: CTE II significant compared to control, CTE I and CTEIV; IGLL5: CTE II significant compared to control and CTE IV). CTE III cases were borderline significant for both IGHM and IGLL5 in pairwise comparison versus control

Correlational network analysis (WGCNA) has previously been used to characterize systems biology changes in both transcriptomic and proteomic studies of neurodegenerative disorders [11, 12, 28–30]. This approach generates modules or groups of genes/proteins with correlated abundance levels in the samples. These related proteins may be associated with specific cell subtypes, biological processes or other novel associations. Indeed, some proteins can even be identified as drivers or hubs of the modules due to increased connectivity with the module members. Modules may also form due to specific traits within the samples like disease, stage or other clinical characteristics. Although network analysis was recently performed on the CTE transcriptome [19, 20], we hypothesized that additional findings may be identified in the CTE proteome, especially when examining CTE cases across the CTE stages I-IV. We included proteins without missing data across the batches, and corrected for covariance with age, region, platform, and batch with ComBat. WGCNA identified 28 modules with clustering via dendrogram tree cutting and the eigengene network presented in Fig. S3. Since PSP and CBD cases were included in the mass spectrometry and the raw data, they were included in the WGCNA to improve power of the analysis. PSP and CBD cases were not however included in the box plots or further analysis because the eigenprotein values for PSP were similar to controls and the number of CBD cases were ultimately too small to draw conclusions. For the control, CTE, and FTLD-MAPT cases, some modules did not associate with any disease related changes (M13-salmon); Fig. 2) whereas others contained interesting co-expression patterns for the eigenprotein values. Representative modules from the 28 modules (Fig. S4) are highlighted in Fig. 2**.** M10-purple demonstrated increases in the eigenprotein value in CTE cases with a stark elevation in the FTLD-MAPT cases as compared to controls. Interestingly, the CTE stages did not appear to show a linear increase from stage I to stage IV (i.e., stage III demonstrated a slight dip as compared to CTE II). The cause of this is unclear but may be related to sampling variability. Other modules, including M1-turquoise, demonstrated a steady drop in the eigenprotein value across CTE stages. The M28-skyblue module showed higher values in the early CTE cases (II and III) but then tapered off in CTE IV and FTLD-MAPT. Finally, modules like M20-royalblue demonstrated an early increase in the eigenprotein value that persisted, with again a notable increase in the FTLD-MAPT cases. The top 8 proteins (represented by gene symbol) contributing the most to the first principal component of each module (i.e., module eigenprotein) are listed under the modules (Fig. 2).

Of these modules, M28-skyblue demonstrated a CTE specific phenotype and this module was enriched with immunoglobulin fragments. Therefore, we used protein blotting to validate two of the top targets (IGHM and IGLL5) and these findings confirmed the increase of immunoglobulin fragments as observed in the module level data (Fig. 2). Similarly, the M23-darkturquoise module was also more specific for CTE, demonstrating increased eigenprotein values across the CTE cases, even CTE I. Gene ontology analysis identified prominent enrichment of collagens and other extracellular matrix proteins in the M23-darkturquoise module (Fig. S6). Finally, modules like M10-purple and M1-turquoise are representative of 17 out of the 28 modules (57.8%; 3884/6713 proteins) that demonstrated that the CTE stages sit on a co-expression continuum between control and FTLD-MAPT cases (Fig. S4). Findings suggest that some aspects of FTLD-MAPT systems biology may serve as an exaggerated phenotype that would be useful for modeling similar disease-related changes in CTE.

### Proteomic neurodegeneration-associated modules are preserved in CTE cases, including significant changes in cell subtype-specific modules

**Fig. 3:**
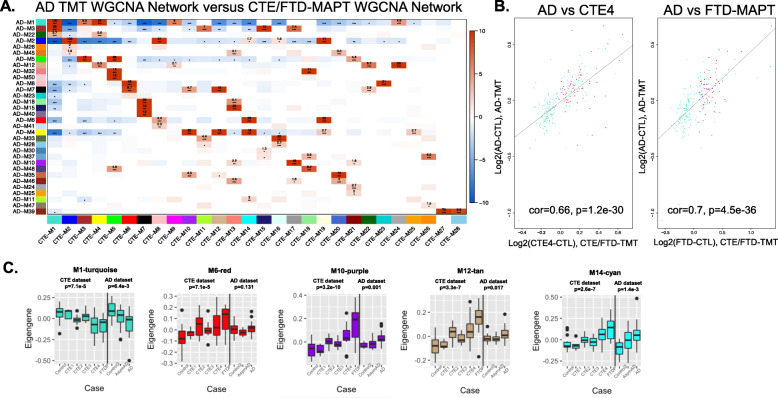
**CTE/FTLD-MAPT TMT WGCNA modules map to AD-TMT modules and demonstrate strong overlap at the individual protein expression level**. **A**. WGCNA module membership was compared between the CTE/FTD TMT proteomic data and recently publish AD TMT dataset. Although module color varied, many of the modules were well preserved between the two datasets. log10 (p-values) are shown in the red and blue boxes which highlight the intersection of modules with statistically significant overlap (red) or depletion (blue). **B**. Correlation at the protein level of log2 expression values demonstrated strong overlap between both AD and CTE IV (left) as well as AD and FTD-MAP (right). Individual proteins are plotted in their respective module colors. Correlation constants and p-values are designated within each plot. **C**. We calculated synthetic eigengenes within the CTE dataset for proteins from the AD dataset. 5 modules identified in Fig. 3B are shown. The vertical line on each box plot separates the two data sets (CTE eigengenes on the left, AD synthetic eigengenes on the right). Control or disease state is listed on the x-axis. Kruskal-Wallis p-values are shown for each group

**Fig. 4:**
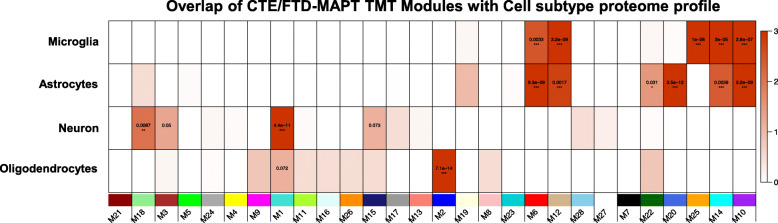
**CTE/FTLD-MAPT TMT modules are enriched with specific cell subtype proteins**. We compared the CTE/FTD TMT proteome to the well-known Sharma cell subtype proteome [24]. Fisher’s exact test with Benjamini Hochberg correction was utilized. Module number and colors are shown across the bottom of the figure and cell subtype on the left. p-values are shown in the red boxes on the heat map. Darker red color indicates a stronger level of significance for a module to be enriched with a particular cell subtype from the brain

Since previous studies demonstrated neuropathological and transcriptomic overlap between CTE and AD [19, 20], we hypothesized that WGCNA would identify modules that were well preserved across the two neurodegenerative diseases. We performed hypergeometric overlap and correlational analysis between this CTE proteomic data set (which included control, CTE and other tauopathies) and the recently published dataset examining AD [22] (which included control, AsymAD and AD; Fig. 3). This AD proteomic dataset was selected as a direct comparator because it was generated using similar methods including TMT labeling and fractionation/liquid chromatography prior to mass spectrometry. There was strong overlap and correlation between the turquoise module members (AD-M1 and CTE-M1), and this module demonstrated steady reduction in the eigenprotein value across serial CTE stages. Cell subtype overlap of CTE network modules using the Sharma reference proteome [24] (Fig. 4) identified prominent enrichment of neuronal components in M1-turquoise, suggesting that the M1-turquoise module is a measure of the neurodegeneration that occurs in CTE, AD, FTLD-MAPT and other neurodegenerative disorders. There was also significant overlap and preservation of modules enriched with glial cells (Figs. 3, 4). These included microglia (AD-M4-yellow mapping to CTE M10-purple, astrocytes (AD M7-black and M8-pink mapping to CTE-M6-red; AD-M35-lightbrown mapping to CTE-M20-royalblue), and oligodendrocytes (AD-M2-blue) mapping to CTE-M2-blue). As in the AD data set (M4-yellow, M7-black and M8-pink) [22], several modules were enriched with multiple cell types in the CTE data set (M6-red, M10-purple, M12-tan, and M14-cyan). Interestingly, comparison of this CTE data set to transcriptome level cell subtype data [25] identified similar cell subtype enrichment in specific modules (neuron for M1-turquoise, microglia for M10-purple, astrocyte for M2-royalblue, oligodendrocyte for M2-blue), as well as additional cell subtype enrichment findings (endothelial for M6-red and M12-tan; oligodendrocyte precursor cells for M23-darkturquoise). When examining individual protein expression levels within the modules, there was a strong correlation between AD and CTE as well as AD and FTLD-MAPT (Fig. 3B) particularly within the M1-turquoise, M14-cyan, M6-red, M10-purple and M12-tan modules. This confirmed that both CTE and FTD were independently contributing to the observed neurodegeneration phenotype (decrease in neuronal expression; increase in glial expression). To provide further clarity on module overlap between the CTE and AD datasets, we also generated synthetic eigengenes for the AD proteins within the CTE modules (Fig. 3C). The eigengene direction of change is comparable (i.e. tracking down in M1-turquoise or up in M10-purple with increasing disease stage) and statistically significant for the AD proteins in 4 of the 5 CTE modules that demonstrated strong correlational overlap in Fig. 3B. There were modules, like the M6-red, in which the AD synthetic eigengenes were not significant.

### Relative cell subtype abundance levels were similar between CTE and AD, with FTLD-MAPT representing an extreme phenotype for astrocyte abundance

**Fig. 5:**
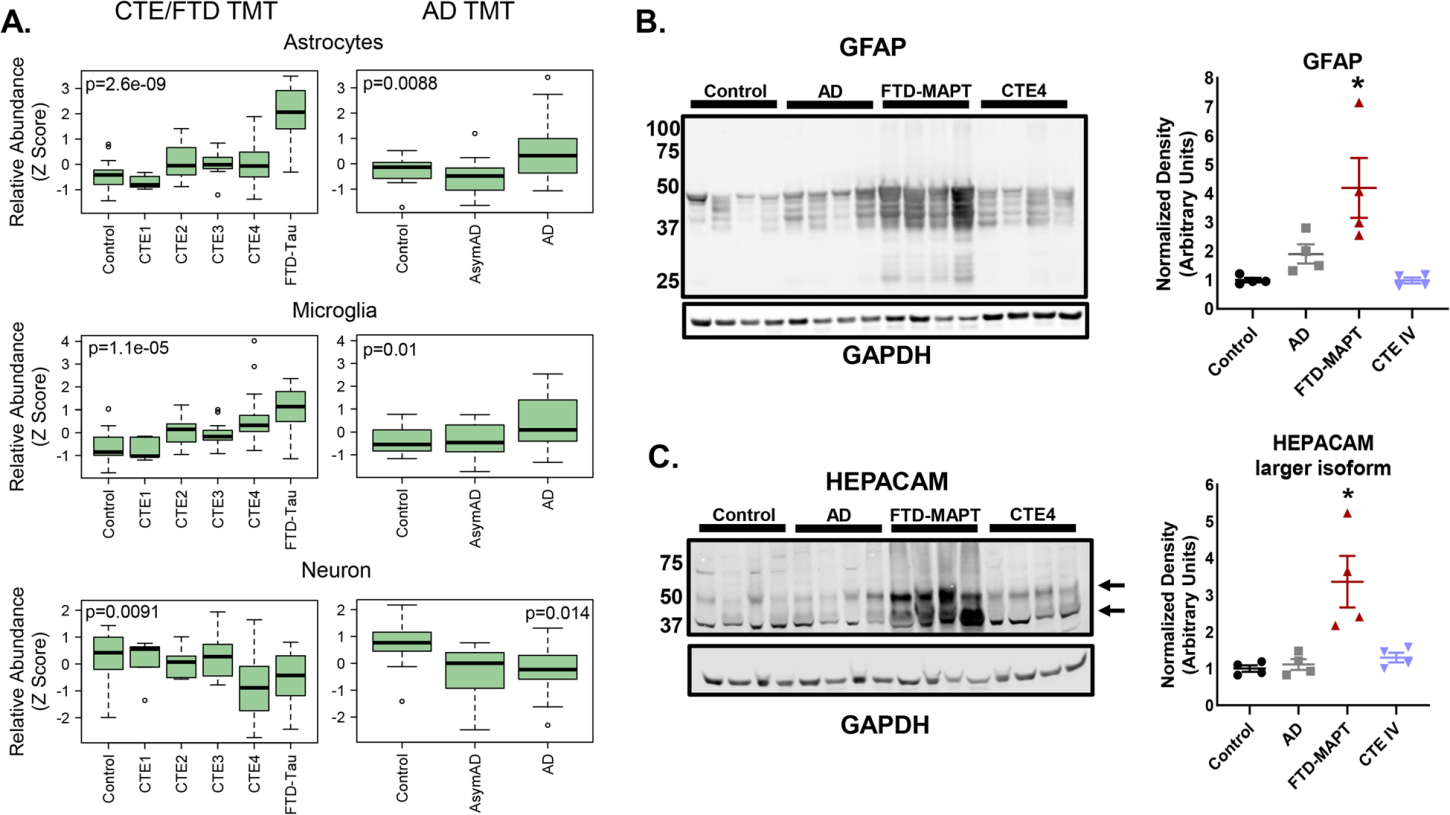
**Cell subtype abundance demonstrated enriched glial proteins with a prominent increase of astrocyte proteins in FTLD-MAPT over that of other tauopathies**. We utilized proteomic protein level data and the Sharma [24] cell type marker classification in combination with the digital sorting algorithm [26] to determine the aggregate abundance of each cell subtype. The CTE/FTD and AD TMT datasets were kept separate, with p-values (Kruskall-Wallis ANOVA) shown for each cell, however relative abundance was normalized via z-score to allow for comparison across datasets. **A**. Box plots of astrocyte, microglia and neuron abundances across control, CTE I-IV and FTLD-MAPT as well as control, asymptomatic AD and AD are shown. Protein blot and densitometry was performed for **B**. GFAP and C. HEPACAM, two astrocyte associated proteins. *designates p-value less than 0.05 (GFAP: FTD-MAPT was significant compared to control and CTE IV; HEPACAM: FTD-MAPT was significant compared to control, AD and CTE IV). As noted in A., astrocyte abundance is greatest in FTLD-MAPT and significantly higher than AD and CTE IV

In addition to the cell subtype analysis in Fig. 4 and Fig. S5, we also determined the relative abundance of cell subtypes across disease states to identify disease-specific findings (Fig. 5). As above, we compared cell type marker-based relative abundance estimates in our CTE proteomic data set to a recently published AD data set which included overlapping controls, asymptomatic AD ((AsymAD) cases with pathology but absent clinical symptoms) and symptomatic AD [22]. Adjacent box plots with z-score normalized abundance levels are displayed (Fig. 5). As observed in the module level-data (Fig. 2) CTE I-IV cases were on the continuum between control and FTD cases for all of the cell subtypes and similar in expression to AD cases. Neuronal loss seems equivalent and slightly greater in CTE IV cases as compared to FTD and AD. Interestingly, CTE II demonstrated similar astrocyte abundance levels to later stages CTE III and IV, suggesting that astrocyte changes occur early in the pathologic cascade and are sustained with increasing neuropathology. This is different from the AsymAD (asymptomatic AD; AD pathology but absent clinical symptoms) to AD comparison, where astrocyte abundance does not seem to increase early. In FTLD-MAPT cases, astrocyte abundance was almost two-fold higher than late stage CTE IV and AD. To confirm this finding, we used protein blotting for two astrocyte-associated proteins: glial fibrillary acidic protein (GFAP) and hepatic and glial cell adhesion molecule (HEPACAM) (Fig. 5B). Both proteins demonstrated significant enrichment in FTLD-MAPT cases as compared to AD and CTE IV, which is consistent with both the aggregate astrocyte abundance (Fig. 5A) and module level data (Fig. 2B**;** M20-royalblue). Findings suggest that FTLD-MAPT has an extreme astrocyte phenotype, although it is unclear if this represents a nonspecific inflammatory signature of FTD, a protective mechanism, or a driver of disease pathogenesis.

## Discussion

In this study, mass spectrometry and bioinformatics was used to provide novel insights across the progressive stages of CTE neuropathology in human brain. CTE disease-related changes were compared to other tauopathies, including FTLD-MAPT and AD. There was strong module preservation between CTE and other neurodegenerative diseases, including modules indicating neurodegeneration, inflammation and glial cell activation/proliferation. The CTE proteome also sits on the tauopathy continuum between control and FTLD-MAPT while increasing CTE stages demonstrated progressive molecular phenotypes in multiple WGCNA modules. Interestingly, the M28-skyblue module and M23-darkturquoise modules were specific for CTE and demonstrated early enrichment of immunoglobulins and extracellular matrix proteins, respectively, suggesting that immunoglobulins and ECM proteins may play roles in early disease pathogenesis. Similarly, astrocyte protein modules also demonstrated early enrichment, highlighting that glial cells are involved early and are sustained in the disease cascade and not just a late-stage epiphenomenon. Overall, this study demonstrated that LC/MS-MS of human brain from increasing stages of CTE can provide novel insights into CTE and identify overlapping changes of CTE with other tauopathies.

A significant finding from this study is that the CTE proteome maintained a neurodegeneration phenotype as previously observed in AD and other neurodegenerative disorders [12, 22, 27]. WGCNA profiling identified multiple overlapping modules with similar directions of change. The M1-turquoise module, which contained multiple neuronal proteins, showed a clear decline in protein expression with increasing CTE stage. In fact, CTE IV cases had similar average expression to that of FTLD-MAPT (Fig. 2), indicating that the neurodegeneration is likely quite prominent in late stage CTE. Multiple glial modules (M10-purple, M20-royalblue, M6-red, M14-cyan, M12-tan) were also identified and demonstrated increased expression profiles, suggesting activation or increased inflammation as a component of CTE disease pathogenesis. Comparison to AD demonstrated prominent module preservation between CTE and AD with respect to module membership and function as well as similar cell subtype abundance. However, when looking at the tauopathy continuum, the CTE stages, including late stage CTE IV, fell below the increases seen for FTLD-MAPT levels in multiple modules and cell subtype abundance, especially with astrocytes, suggesting that FTLD-MAPT has a more prominent glial component. When compared to other published findings, this neurodegeneration phenotype was also observed in the CTE transcriptome [19], confirming some preservation of and consistency among the transcriptome and proteome, a relationship that is not always observed. Finally, multiple modules demonstrated that the eigenprotein values of CTE cases sit between control and FTLD-MAPT. Since these are static time points of convenience from a cross-sectional cohort, CTE on this continuum does not imply a clear linear association between the tauopathies. Similarly, FTLD-MAPT is not an outright model for CTE disease pathogenesis, however, some of the systems biology findings could be useful for studying aspects of CTE. This may be especially applicable in modules where the eigenprotein has increasing values with increasing CTE stage where FTLD-MAPT also has a similar direction of change.

In addition to WGCNA, differential expression versus control was used to identify individual proteins and pathways. This approach was limited in CTE I-III due to lower numbers of significant proteins, but in CTE IV well over 1000 differentially expressed proteins were identified. Comparison to AD and FTLD-MAPT demonstrated more prominent overlap of CTE IV with FTLD which is an interesting finding. Although co-pathologies have been described in CTE IV cases (i.e., TDP-43, amyloid), this significant overlap with the FTLD-MAPT continues to highlight the close relationship between the two pure tauopathies. Furthermore, 167 proteins demonstrated significant expression across all 3 disease states (CTE IV, AD, and FTLD-MAPT). Gene ontology suggested that this group contained a mix of cytoskeleton, transport, and cell projection proteins as well as neuronal markers. Of note, a previous study evaluating the proteome of a small sample of CTE IV cases identified changes in axonal guidance, thus showing some validation and similarity with the cell projection proteins in this dataset [18]. Interestingly, the overlap between that study and the CTE IV cases in this study was quite limited with only 244 proteins out of the 1371 significant proteins that were identified. In addition, the direction of change was also not consistent between the overlapping 244 proteins. Almost 10% were increased in our dataset but decreased in the other, whereas 25% were decreased in our dataset but increased in the other. These variances may be accounted for by sample variability and preparation, case and control selection, and brain regional differences.

We also compared the CTE total proteome to our previously published CTE insoluble proteome. Over 4600 proteins were common to both data sets with only 45 demonstrating statistical significance in both. 16/45 localized to the M1-turquoise module with 29/45 spread across multiple other modules. The most prominent of the proteins included ITM2B, NQO1, CRYZ, C4A and SQSTM1, which have all been described in some context with neurodegenerative disorders. The NQO1 finding is especially interesting given the prominent association with tau pathology and astrocytes and that astrocyte changes are up early and sustained in the CTE neuropathological cascade. The increase in NQO1 in the total proteome suggests a protective response by the brain to manage reactive oxygen species, whereas an increase in the insoluble proteome could be deleterious (i.e. working under the assumption that insoluble proteins may be less functional due to their insoluble/aggregated state).

The CTE proteome, like CTE neuropathological staging, appears to have unique molecular profiles that evolve over time. In most cases, CTE stage I had protein expression levels nearly identical to controls (Figs. 2, 3, 5, S2), which is not surprising given the relatively paucity of p-tau pathology. One exception to this however was in the M23-darkturquoise module where CTE stage I had similar aggregate expression to the other CTE stages (Fig. 2). The M23-darkturquoise module is significantly enriched with ECM proteins, suggesting that early disease-related changes occur in CTE I prior to evidence for neurodegeneration and glial upregulation and/or proliferation. CTE II-IV, however, demonstrated neuronal loss and increased expression of glial proteins. Although a linear decrease or increase (depending on the module; neuronal or glial respectively) across the CTE stages was expected, CTE stage III demonstrated a slight decrease in the eigenprotein value as compared to CTE stages II and IV. The cause for this is unclear but might be due to sampling variability, which like in CTE I, may still be present even in later stage cases (i.e., despite more widespread p-tau, there could still be areas of brain that were sampled with lower levels of pathology given the patchy nature of CTE).

In addition to identifying progression across the CTE stages in modules, there were also modules uniquely associated with CTE, like the M28-skyblue module. This module demonstrated little change in CTE stage I but a sharp increase with CTE stage II followed by a slow decline through CTE stages III and IV. Interestingly, this module contained numerous immunoglobulins, both heavy and light chains, which was confirmed by immunoblotting (Fig. 2). These findings suggest that immunoglobulins breach the blood brain barrier in early CTE stages, a finding supported by neuropathological evidence of microvascular disruption [31]. It is unclear if this represents an indirect measure of endothelial disfunction, as we also observed changes in endothelial cells in the red module, and/or if these immunoglobulins may actually be contributing to the disease process. Mechanisms could include either autoimmunity or microvascular disruptions of the blood brain barrier due to repetitive traumatic injury [32]. Autoimmune antibody-mediated encephalopathies may also be associated with brain injury [33, 34]. Future studies are needed to understand the contribution of immunoglobulins to CTE pathogenesis.

One surprising finding is that we did not identify enrichment of expected CTE neuropathology associated proteins. Given the robust nature of tau pathology in both CTE IV and FTLD-MAPT cases, we expected that MAPT would serve as a positive control for the experiment based on previous findings of MAPT enrichment in the CTE insoluble proteome. MAPT was identified and localized to neuronal modules (M1-turquoise and M9-magenta), but did not demonstrate significant enrichment with differential expression or high kME values. Indeed as noted above, the eigengene in those modules actually goes down and not up. This was similar for TARDBP (gene symbol for TDP43) which is another protein that can abnormally accumulate in CTE. It localized to the M7-black module which is enriched with RNA binding and nuclear proteins and has an eigenprotein value that goes up with disease, but TARDBP did not demonstrate significant enrichment based on differential expression. Of note, we have observed this issue in other proteomic datasets, including with AD where proteins that aggregate are significant in the insoluble proteomes but not the total proteomes. Amyloid pathology has also been described in later stage CTE cases [14], however, the CTE cases selected for this study did not have a significant amyloid pathology burden.

Finally, the prominent FTLD-MAPT astrocyte abundance was an unexpected finding. Although both CTE and AD demonstrated similar increased abundance of both astrocyte and microglia proteins, FTLD-MAPT astrocyte expression was notably further elevated. Astrocytes play multiple roles in the brain, including maintenance of the ECM, synapses, connection to vasculature and the glymphatic system. Although this could be secondary to a more robust inflammatory response in FTLD-MAPT, astrocytes in many tauopathies, including CTE, FTD, AD, PSP, CBD, and others [35–40], accumulate tau pathology. Given that FTLD-MAPT is a pure tauopathy with underlying genetic mutations in MAPT, the robust astrocyte phenotype and pathological accumulation of tau in astrocytes might suggest additional mechanisms are contributing to disease pathogenesis.

There were several limitations to this study including limited tissue samples of individual brains, low case number and the cross-sectional nature of sample collection. Bayesian inference (ComBat) and regression analysis were used to remove possible confounders, however, as with any human brain study, there is always heterogeneity that may limit our ability to detect certain disease related changes in one cohort versus another. Comparison to other data sets, either transcriptomic or proteomic, can also be challenging given that multiple variables are likely different (case samples, machines, biochemical isolation techniques). Additional prospective studies as well as in vivo biomarkers (i.e., plasma, cerebrospinal fluid) will help refine correlational analyses as new tools become available in the future.

## Conclusions

These novel findings broaden our understanding of protein and molecular changes that occur in early CTE and across the CTE progression, including CTE specific changes in the M23-darkturquoise ECM and M28-skyblue immunoglobulin modules. The strong overlap of CTE proteomic changes with those in FTLD-MAPT suggests that FTLD-MAPT and model systems may be useful for studying some aspects of CTE pathogenesis, particularly in those modules where the CTE eigenprotein is on a continuum between control and FTLD-MAPT. Finally, this TMT proteomic dataset will serve as an outstanding resource for future studies into CTE, FTD, AD and other tauopathies.

## Supplementary Information

**Additional file 1 **Fig. S1. Violin plots to visualize covariance before (left) and after (right) Combat. Plots were generated using the variancePartition package in R. Region, Batch and Platform covarying distribution tail proteins are indeed improved. Fig. S2. Volcano plots of proteomic data for tauopathies versus control. -log10 (*p*-value) is plotted against the log2 abundance of CTE I, CTE II, CTEIII, CTE IV, FTLD-MAPT and AD minus control abundance. Red dots are below the *p* < 0.05 cutoff (significant) whereas black dots are above the cutoff (not significant). *p*-values determined by ANOVA with post-hoc Tukey’s HSD with built-in correction for multiple comparisons. Fig. S3. Cluster dendrogram and Eigengene Network for CTE/FTD TMT WGCNA network. Traits are listed under the Cluster dendrogram and module colors following regression. The Eigengene network demonstrates a high-level view of relatedness between the different modules. For example, the M10-purple and M14-cyan as well as M6-red and M12-tan modules are strongly related as demonstrated in the hierarchical tree and in the correlational heat map below. Fig. S4. Box plots for modules from CTE/FTD TMT WGCNA network. Kruskal Wallis *p*-values and number of proteins (n) are shown for each of the modules. * designates modules that are both significant with p-value < 0.05 and demonstrated either increase or decrease in the eigengene between controls and FTLD-MAPT with CTE cases demonstrating an intermediate phenotype on the continuum. Fig. S5. CTE/FTLD-MAPT TMT modules are enriched with specific cell subtype proteins. We compared the CTE/FTD TMT proteome to the Barres cell subtype mouse transcriptome database. Fisher’s exact test with Benjamini-Hochberg correction was utilized. Module number and colors are shown across the bottom of the figure and cell subtype on the left. p-values are shown in the red boxes on the heat map. Darker red color indicates a stronger level of significance for a module to be enriched with a particular cell subtype from the brain. Comparison to the rodent transcriptome identified additional neuronal modules and also highlighted modules with other possible cell types including endothelial cells (M6-red and M12-tan modules) and oligodendrocyte precursor cells (M23-darkturquoise). Fig. S6. Gene ontology graphs for all 28 modules. (PPTX 9669 kb)

**Additional file 2.** Table S1. Demographics and traits: Cases are grouped according to diagnosis. Case number is either Emory (letter followed by numbers) or Boston University (4-digit number). Age, Sex, PMI (postmortem interval), and brain region are shown for each case. Batch indicates the TMT batch numbers (1–10). Channel within batch is listed in 2 columns, both as a single number and as a 3-digit number with letter. Platform designates which mass spectrometer was utilized, either the Fusion 1 or Lumos. Demographic summary chart is also included. Table S2. ANOVA output for CTE/FTLD-MAPT dataset: ANOVA data for 6713 proteins without missing values across 10 batches. All combinations are shown between control and neurodegenerative diseases including p-values and differential expression. Corresponding data from the AD TMT dataset for each of the proteins is also included in columns BK and BJ. Table S3. Significantly enriched proteins identified in AD, CTE 4 and FTLD-MAPT and Gene Ontology for Biological Process, Molecular Function and Cellular Component. Table S4. kME Values for proteins within each module following WGCNA of the CTE/FTLD-MAPT TMT dataset. Table S5. Raw abundance data from CTE/FTLD-MAPT TMT Proteome. Table S6. WGCNA module number, color and size.

## Data Availability

The datasets generated during the current study are available in Synapse (www.synapse.org; Synapse ID: syn11612204). The mass spectrometry data have also been deposited to the ProteomeXchange Consortium via the PRIDE partner repository (https://www.ebi.ac.uk/pride/) with the dataset identifier PXD024724. Demographics and data summaries are also included in Table S1-S6**.**

## Abbreviations

AD: Alzheimer’s disease
ANOVA: analysis of variance
AsymAD: asymptomatic Alzheimer’s disease
BCA: bicinchonic acid
C4A: complement C4A
CBD: corticobasal degeneration
CRYZ: crystallin, zeta; quinone oxidoreductase
CTE: chronic traumatic encephalopathy
DNA: deoxyribonucleic acid
DSA: digital sorting algorithm
ECM: extracellular matrix
FTLD-MAPT: frontotemporal dementia due to microtubule associated protein tau mutations
GFAP: glial fibrillary acidic protein
GIS: global internal standard
HEPACAM: hepatic and glial cell adhesion molecule
IGHM: immunoglobulin heavy constant mu
IGLL5: immunoglobulin lambda like polypeptide 5
IRB: Institutional Review Board
ITM2B: integral membrane protein 2B
LC-MS/MS: liquid chromatography and tandem mass spectrometry
MAPT: microtubule associated protein tau
NQO1: NADPH quinone oxidoreductase
PMI: postmortem interval
PSP: progressive supranuclear palsy
RHI: repetitive head impacts
RNA: ribonucleic acid
SD: standard deviation
SDS-PAGE: sodium dodecyl sulfate polyacrylamide gel electrophoresis
SQSTM1: sequestosome-1
TBI: traumatic brain injury
TBS: Tris buffered saline
TDP43: TAR DNA binding protein 43
TMT: tandem mass tagged
WGCNA: weighted gene co-expression network analysis; correlation network analysis
