# Supplementary material for: A proteomic network approach resolves stage-specific molecular phenotypes in chronic traumatic encephalopathy
Source: Mol Neurodegener. 2021 Jun 25;16:40. doi: 10.1186/s13024-021-00462-3 (PMC8235576; doi:10.1186/s13024-021-00462-3)
Supplement: Supplementary file 1 — Additional file 1 Fig. S1. Violin plots to visualize covariance before (left) and after (right) Combat. Plots were generated using the variancePartition package in R. Region, Batch and Platform covarying distribution tail proteins are indeed improved. Fig. S2. Volcano plots of proteomic data for tauopathies versus control. -log10 (p-value) is plotted against the log2 abundance of CTE I, CTE II, CTEIII, CTE IV, FTLD-MAPT and AD minus control abundance. Red dots are below the p < 0.05 cutoff (significant) whereas black dots are above the cutoff (not significant). p-values determined by ANOVA with post-hoc Tukey’s HSD with built-in correction for multiple comparisons. Fig. S3. Cluster dendrogram and Eigengene Network for CTE/FTD TMT WGCNA network. Traits are listed under the Cluster dendrogram and module colors following regression. The Eigengene network demonstrates a high-level view of relatedness between the different modules. For example, the M10-purple and M14-cyan as well as M6-red and M12-tan modules are strongly related as demonstrated in the hierarchical tree and in the correlational heat map below. Fig. S4. Box plots for modules from CTE/FTD TMT WGCNA network. Kruskal Wallis p-values and number of proteins (n) are shown for each of the modules. * designates modules that are both significant with p-value < 0.05 and demonstrated either increase or decrease in the eigengene between controls and FTLD-MAPT with CTE cases demonstrating an intermediate phenotype on the continuum. Fig. S5. CTE/FTLD-MAPT TMT modules are enriched with specific cell subtype proteins. We compared the CTE/FTD TMT proteome to the Barres cell subtype mouse transcriptome database. Fisher’s exact test with Benjamini-Hochberg correction was utilized. Module number and colors are shown across the bottom of the figure and cell subtype on the left. p-values are shown in the red boxes on the heat map. Darker red color indicates a stronger level of significance for a module to be enriched with a pa [file 13024_2021_462_MOESM1_ESM.pptx]

## Slide 1
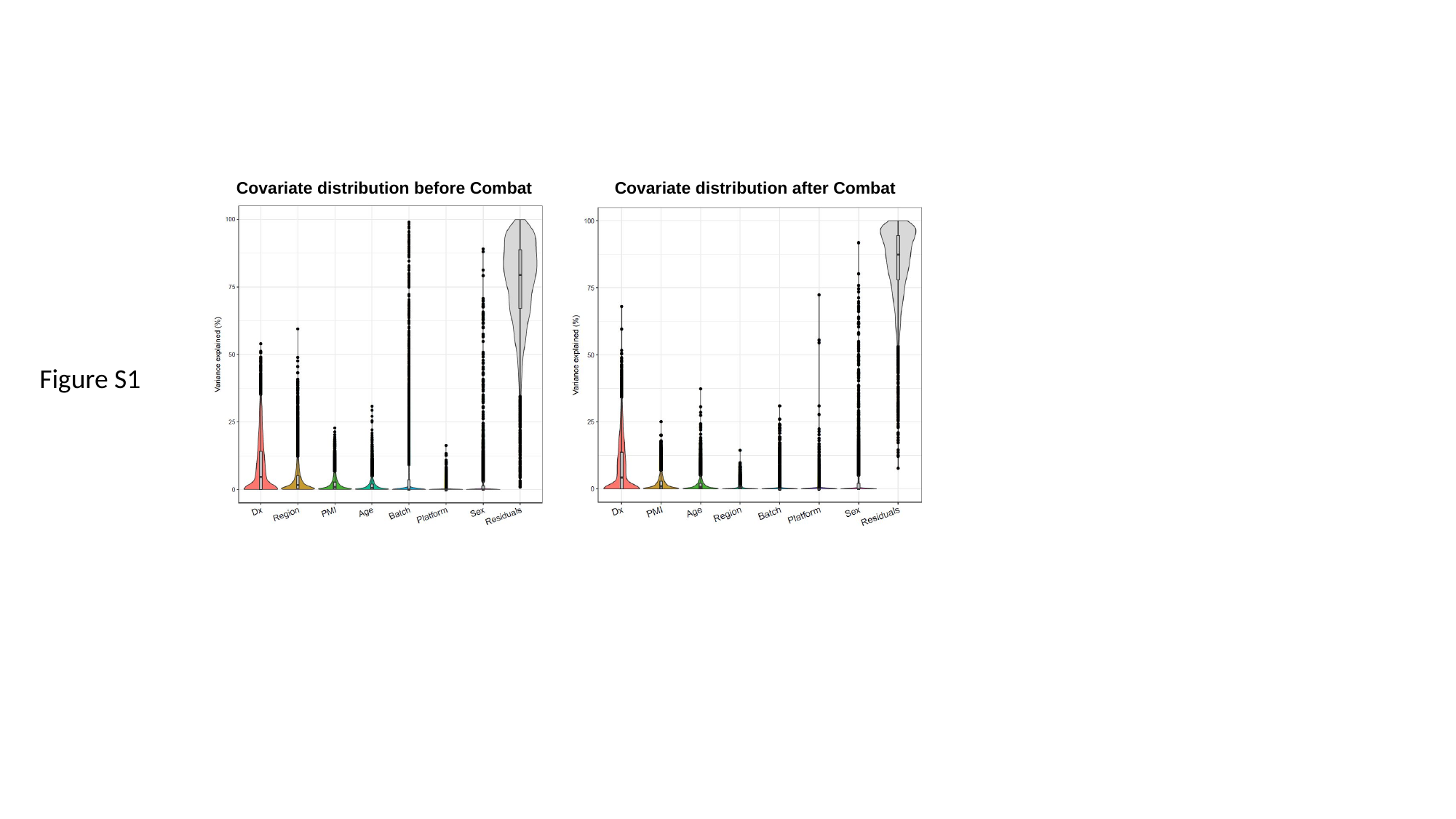

Covariate distribution before Combat
Covariate distribution after Combat
Figure S1

## Slide 2
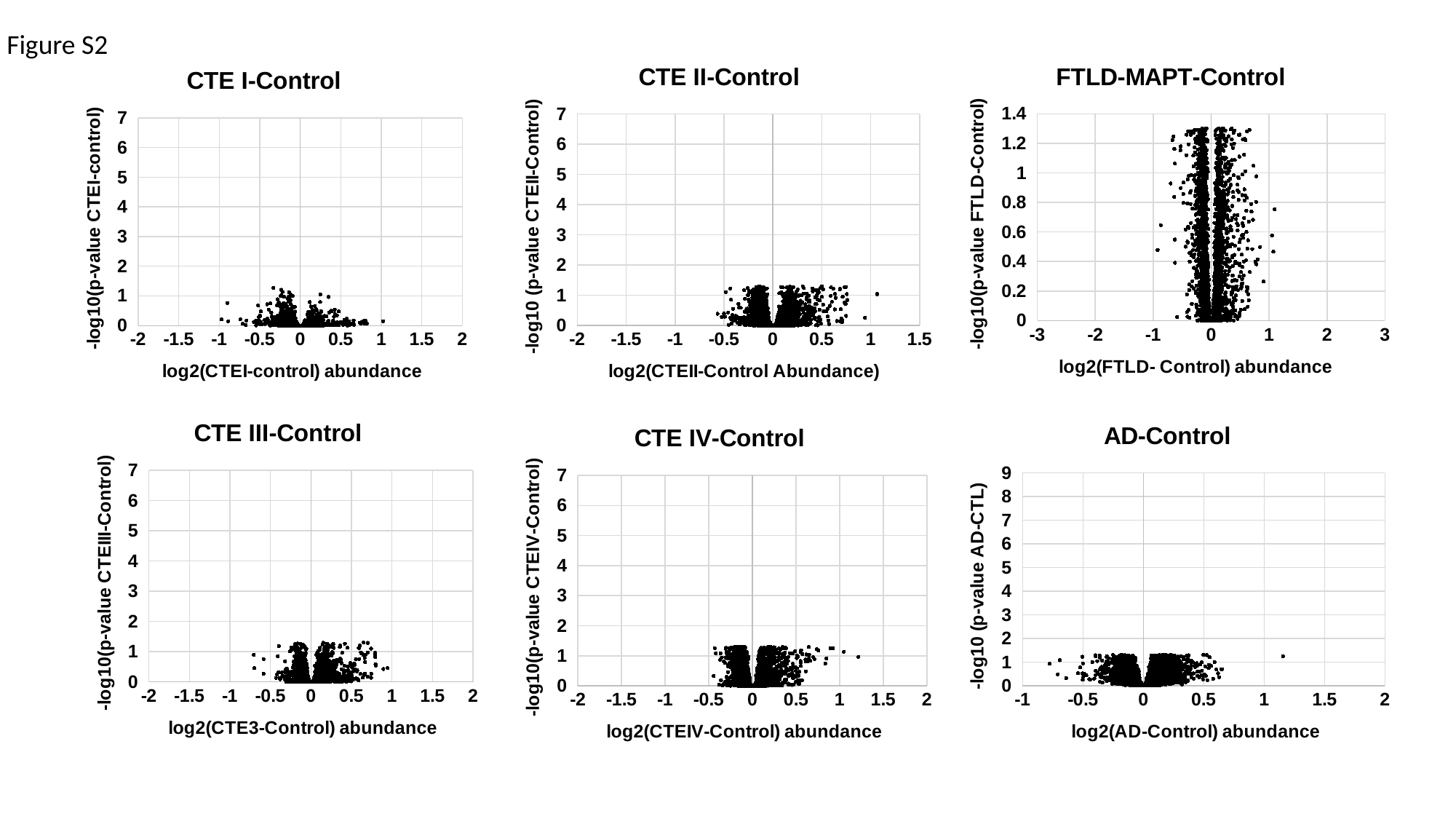

Figure S2
### Chart: FTLD-MAPT-Control
| Category | | |
|---|---|---|
### Chart: CTE II-Control
| Category | | |
|---|---|---|
### Chart: CTE I-Control
| Category | CTE1-CTL | |
|---|---|---|
### Chart: CTE III-Control
| Category | | |
|---|---|---|
### Chart: AD-Control
| Category | | |
|---|---|---|
### Chart: CTE IV-Control
| Category | | |
|---|---|---|

## Slide 3
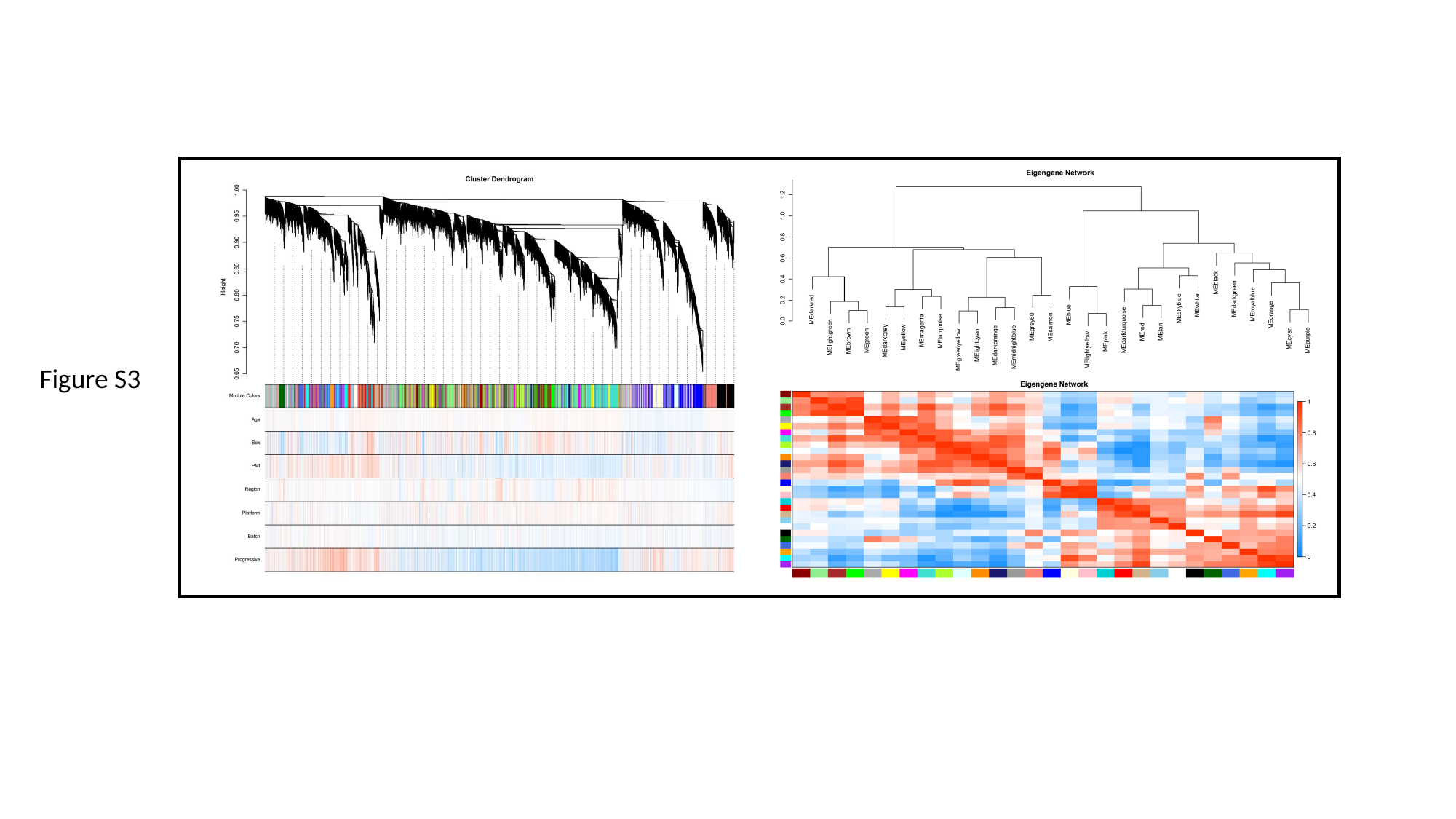

Figure S3

## Slide 4
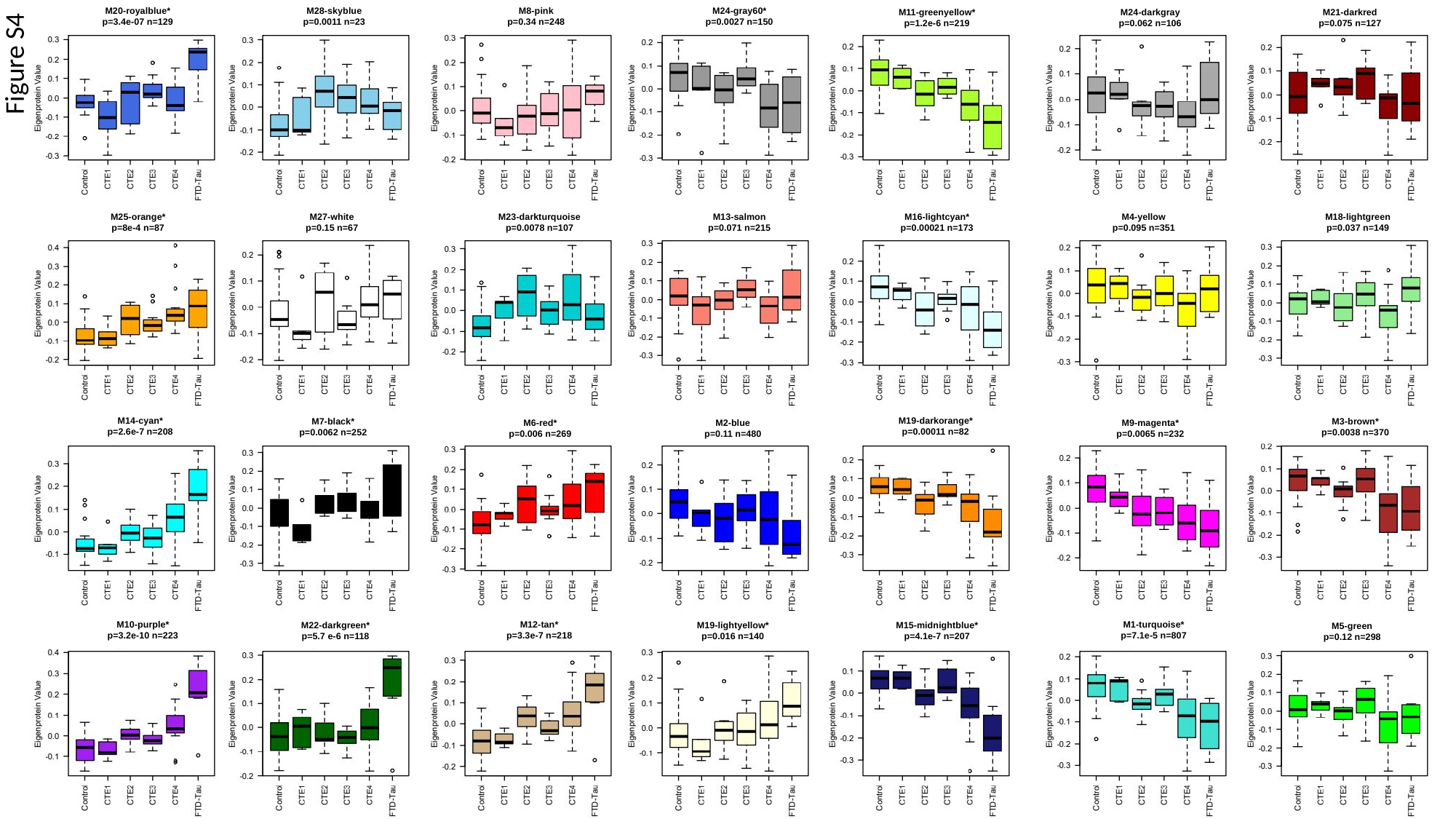

M20-royalblue*
p=3.4e-07 n=129
M8-pink
p=0.34 n=248
M24-gray60*
p=0.0027 n=150
M28-skyblue
p=0.0011 n=23
M11-greenyellow*
p=1.2e-6 n=219
M24-darkgray
p=0.062 n=106
M21-darkred
p=0.075 n=127
Figure S4
M25-orange*
p=8e-4 n=87
M27-white
p=0.15 n=67
M23-darkturquoise
p=0.0078 n=107
M13-salmon
p=0.071 n=215
M16-lightcyan*
p=0.00021 n=173
M4-yellow
p=0.095 n=351
M18-lightgreen
p=0.037 n=149
M14-cyan*
p=2.6e-7 n=208
M19-darkorange*
p=0.00011 n=82
M7-black*
p=0.0062 n=252
M3-brown*
p=0.0038 n=370
M6-red*
p=0.006 n=269
M2-blue
p=0.11 n=480
M9-magenta*
p=0.0065 n=232
M12-tan*
p=3.3e-7 n=218
M1-turquoise*
p=7.1e-5 n=807
M10-purple*
p=3.2e-10 n=223
M15-midnightblue*
p=4.1e-7 n=207
M19-lightyellow*
p=0.016 n=140
M22-darkgreen*
p=5.7 e-6 n=118
M5-green
p=0.12 n=298

## Slide 5
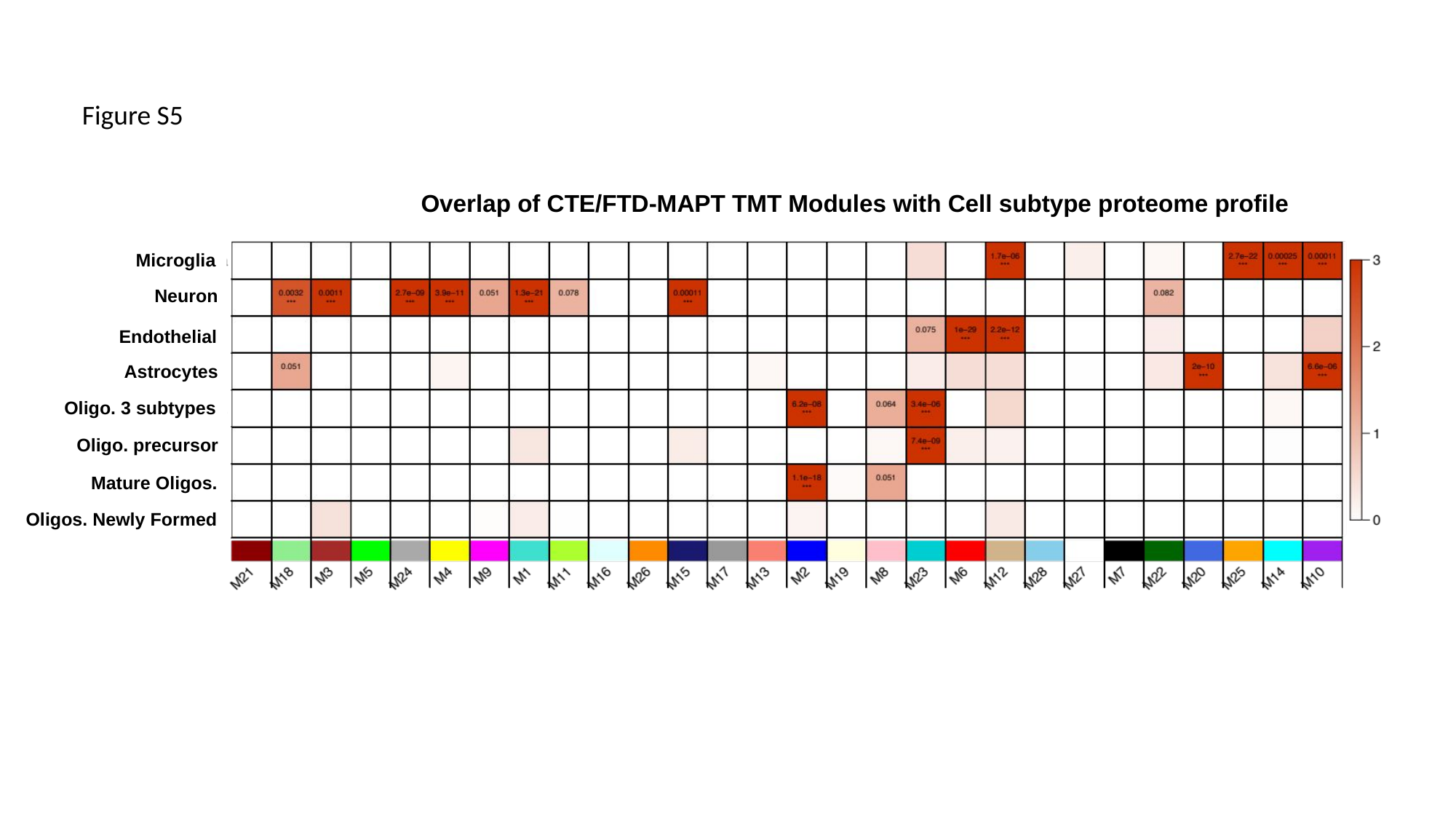

Figure S5
Overlap of CTE/FTD-MAPT TMT Modules with Cell subtype proteome profile
Microglia
Neuron
Endothelial
Astrocytes
Oligo. 3 subtypes
Oligo. precursor
Mature Oligos.
Oligos. Newly Formed

## Slide 6
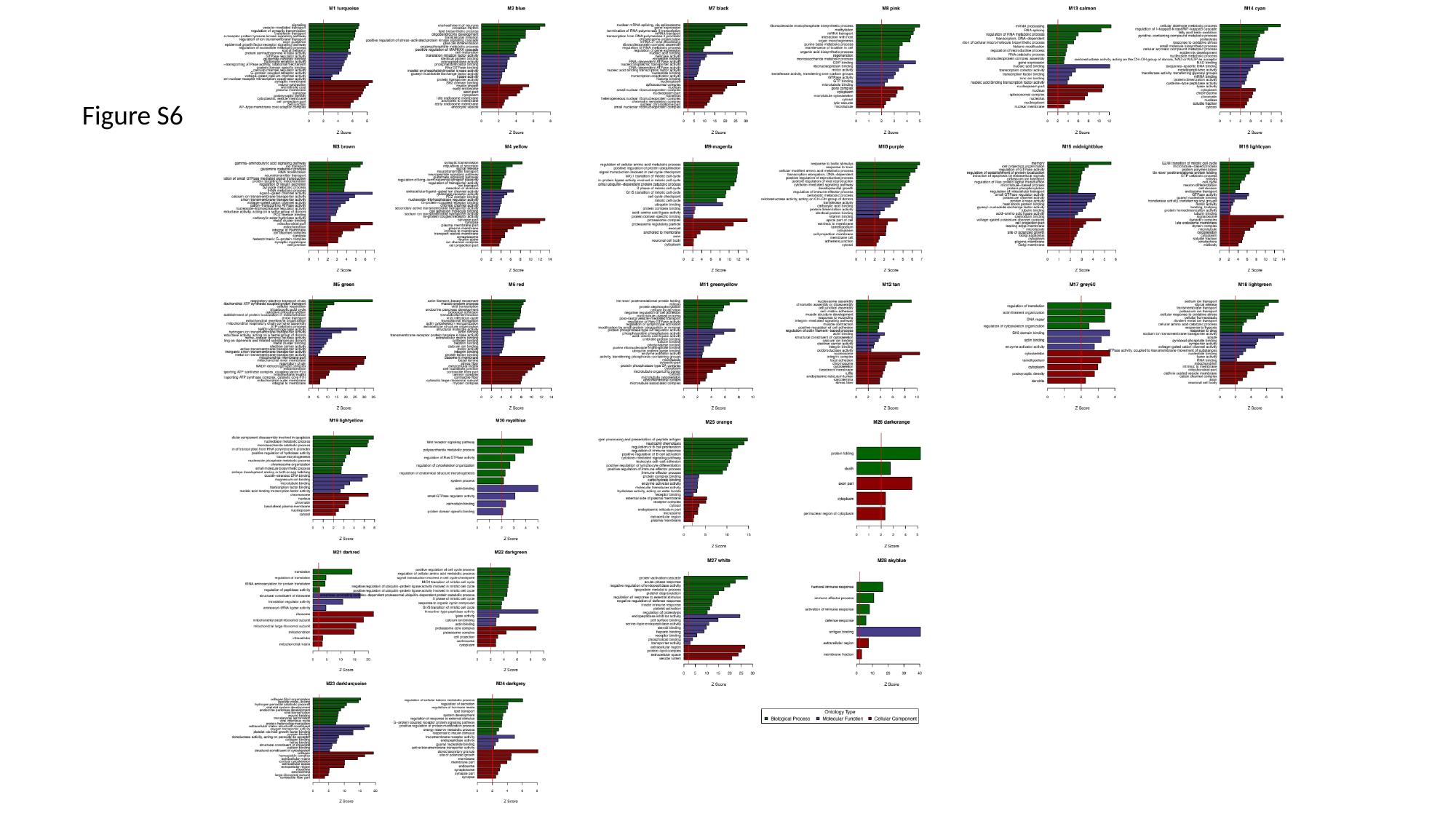

Figure S6
